# Hopelessness as a basis for tuberculosis diagnostic delay in the Arkhangelsk region: a grounded theory study

**DOI:** 10.1186/1471-2458-13-712

**Published:** 2013-08-02

**Authors:** Vladimir N Kuznetsov, Andrej M Grjibovski, Andrej O Mariandyshev, Eva Johansson, Donald A Enarson, Gunnar A Bjune

**Affiliations:** 1 Institute for Health and Society, University of Oslo, Oslo, Norway; 2 Institute of Mental Medicine, Northern State Medical University, Arkhangelsk, Russia; 3 International School of Public Health, Northern State Medical University, Arkhangelsk, Russia; 4 Department of International Public Health, Norwegian Institute of Public Health, Oslo, Norway; 5 Department of Tuberculosis, Northern State Medical University, Arkhangelsk, Russia; 6 Karolinska Institute, Stockholm, Sweden; 7 International Union against Tuberculosis and Lung Diseases, Paris, France; 8 Nordic School of Public Health, Gothenburg, Sweden; 9 Northern State Medical University, Troitskii Ave 51, Arkhangelsk, 163000, Russia

**Keywords:** Diagnostic delay, Tuberculosis, Qualitative research, Grounded theory

## Abstract

**Background:**

Data about delayed tuberculosis diagnosis in Northern Russia are scarce yet such knowledge could enhance the care of tuberculosis. The Arkhangelsk region is situated in the north of Russia, where the population is more than one million residents.

The aim of the study was to understand factors influencing diagnostic delay among patients with tuberculosis in the Arkhangelsk region and to develop a theoretical model in order to explain diagnostic delay from the patients’ perspectives.

**Methods:**

Twenty-three patients who had experienced diagnostic delay of tuberculosis were interviewed in Arkhangelsk. Using a qualitative approach, we conducted focus-group discussions for data gathering using Grounded Theory with the Paradigm Model to analyse the phenomenon of diagnostic delay.

**Results:**

The study resulted in a theoretical model of the pathway of delay of tuberculosis diagnosis in the Arkhangelsk region in answer to the question: “Why and how do patients in the Arkhangelsk region delay tuberculosis diagnosis?” The model included categories of causal conditions, context and intervening conditions, action/interaction strategies, and consequences. The causal condition and main concern of the patients was that they were overpowered by hopelessness. Patients blamed policy, the administrative system, and doctors for their unfortunate life circumstances. This was accompanied by avoidance of health care, denial of their own health situations, and self-treatment. Only a deadly threat was a sufficient motivator for some patients to seek medical help. “Being overpowered by hopelessness” was identified as the core category that affected their self-esteem and influenced their entire lives, including family, work and social relations, and appeared even stronger in association with alcohol use. This category reflected the passive position of many patients in this situation, including their feelings of inability to change anything in their lives, to obtain employment, or to qualify for disability benefits.

**Conclusion:**

The main contributing factor to unsuccessful health-seeking behaviour for patients with tuberculosis was identified as “being overpowered by hopelessness.” This should be taken into consideration when creating any preventive programs and diagnostic algorithms aimed at increasing knowledge about TB, improving the health system, decreasing alcohol consumption and reducing the poverty of the people in Arkhangelsk.

## Background

Diagnostic delay for tuberculosis patients is usually defined as the duration of time from onset of symptoms to the initiation of treatment. It includes patient delay (from onset of symptoms to the first interaction with health services) and systems delay (from the first visit to health services to initiation of treatment) [1,2]. While there are many studies of diagnostic delay internationally [3], data about the situation in Russia are scarce, and such knowledge could enhance the care of tuberculosis.

In the Arkhangelsk region, the incidence of tuberculosis decreased 43.2% from 2000–2011 (from 104 to 40.2 per 100 000) and the death rate dropped during the same period from 16.2 to 6.5 per 100 000 [4]. Nevertheless, a large number of deaths are due to unsuccessful treatment of drug-resistant strains of *Mycobacterium tuberculosis* that have been found in 47% of new cases in Arkhangelsk. These deaths are possibly enhanced by delay in diagnosis and treatment occurs too late for patients to be treated successfully [5-7]. The association between delay and drug resistance may be further promoted by the fact that patients with drug-resistant forms of tuberculosis are more likely to abuse alcohol [8,9]. Delay in diagnosis of multidrug-resistant tuberculosis (MDR-TB) is of particular concern due to the risk of transmission of this form of tuberculosis to others.

Risk factors for diagnostic delay include poverty, low educational level, poor knowledge about tuberculosis, single status, large family size, being a farmer, belonging to an indigenous group, not having health insurance [10,11], rural residence, seeking care from traditional or unqualified practitioners, inappropriate antibiotic treatment [12], old age, immigrant or illegal resident status, female sex [13], and beliefs and attitudes [3,11,14].

Health behaviour is related to general behaviour and behaviour is modifiable. However, an individual is not a rational decision–maker; in reality, decision-making is unpredictable. Consequently, understanding the individual and his/her cognitive processes (‘I know, therefore I act’) is essential [15].

In general, socially disadvantaged groups have specific types of health-seeking behaviour based on their beliefs. They often use self-treatment, visit traditional healers and may seek care from official medical specialists. The recognition and interpretation of symptoms by the patients and their society is a precondition for seeking care from a specialist and for successful diagnosis and treatment [16,17]. Understanding depends on existing views of the likely meaning of the symptoms and the availability and accessibility of various potential sources of assistance (traditional, spiritual, Western medicine) [16,17]. For example, the presence of alternative sources of assistance, coupled with ignorance of the symptoms of TB, stigma, and problems of access to medical facilities led to significant delays in diagnosis and treatment of tuberculosis in Ethiopia [18].

Health seeking behaviour is the result of the interaction between changes in physiological activity, autonomic arousal, affective integration, and the process of cognitive interpretation. This interaction leads through subjective distress to an adaptation behaviour (such as coping). Psychological processes are central and inevitably interact with the biological milieu and sociocultural environment and thus cannot be considered in isolation [19]. Specific personal and social values affect experience of the disease and are usually based on people’s knowledge, beliefs, and ideas about health and disease, including causes, signs and symptoms, severity, transmission, treatment and prognosis [19]. To gain an accurate picture of health-seeking behaviour, we need to pay special attention to the culturally-specific characteristics of patients (their knowledge, beliefs, perceptions, etc.), the context, and the accuracy of data collection methods [19].

The purpose of this study was to improve our understanding of factors influencing diagnostic delay among patients with tuberculosis in Arkhangelsk and to develop a theoretical framework/model to explain this delay from the patients’ perspectives.

## Methods

### Setting

The Arkhangelsk region is situated in the north of Russia with a total area of 589,913 square kilometres. The population is 1,213,533 people (2012) [20,21]. Most people have lived there for a long time and many originated from dissidents deported to this area long ago. The main fields of work are fishing, the timber and pulp industry, and engineering (such as the defence industry) [20-22].

There are seven outpatient and 10 inpatient clinics in Arkhangelsk and 32 health centres (outside of Arkhangelsk) which all refer tuberculosis patients to the Arkhangelsk anti-tuberculosis dispensary for treatment. The dispensary treats both inpatients and outpatients. All patients with multidrug-resistant tuberculosis and patients with sensitive forms of tuberculosis with positive smear microscopy begin anti-tuberculosis treatment in the hospital. The initial phase of treatment takes six to nine months. The continuation phase is provided in outpatient clinics in the place of residence after the smear microscopy becomes negative.

### Study design

Grounded Theory and, in particular, the Paradigm Model of Strauss and Corbin [23,24], was used to explore the phenomenon of diagnostic delay. Grounded Theory is used for the systematic generation of theory from data using both inductive and deductive reasoning. One of the goals of Grounded Theory is to discover the participants’ main concerns in order to understand the processes in which they are involved using empirical research. The researcher also studies how the participants continually try to resolve their concerns regardless of time and place [23,24].

Health perceptions and perceptions concerning tuberculosis services and factors influencing diagnostic delay were discussed by both men and women in focus group discussions (FGDs). The findings of FGDs were used to generate a tentative model based on empirical data. The tentative model allows an exploration of reasons for tuberculosis diagnostic delay from a patient’s perspective [23,25,26].

As this knowledge was limited, FGDs were chosen as an appropriate data collection method given the short timeframe. We also obtained a wide variety of opinions from patients who had the diagnosis of tuberculosis for the first time through guided discussions [27]. The FGDs provided an opportunity to study ideas and concepts used in this cultural context. The method, while providing a diversity of opinions, did not aim to reach consensus or influence the participants in any way [28]. This was a relatively efficient manner to gather data as compared to individual interviews [29]. FGDs encourage participation of patients who perceive individual interviews as too formal and isolated [30] and facilitates discussion of taboo topics. Being among peers gives the group confidence to speak about ideas that differ from the culture of the researcher [29] and offers the opportunity to criticize socially accepted norms [31,32]. Grounded Theory was chosen because we aimed to generate a new theory of reasons for diagnostic delay from the participants’ perspective [23,33].

The research team consisted of researchers with different backgrounds from Russia, Canada, Sweden and Norway: three tuberculosis specialists (D.E., G.B and A.M), one nurse (E.J) with expertise in public health and qualitative research, one epidemiologist (A.G) and one psychiatrist with experience of public health who was not involved in the tuberculosis management (principal investigator, V.K). The investigators brought their own perspectives to the analysis. Two clinical psychologists in the anti-tuberculosis dispensary assisted in the FGDs. Coding was done by the principal investigator and later shared and discussed with the other authors.

### Participants

Eligible patients were adults newly admitted for hospital treatment who had smear-positive sputum. The study included only patients who were new cases with a drug-susceptible form of tuberculosis and who came to a department specializing in cases of tuberculosis with diagnostic delay. Doctors at the hospital, who were not part of the research team, evaluated the information about the diagnostic delay. This evaluation was used as a part of the analysis giving additional information for generation of categories. Recruitment for the FGDs was done by doctors who did not take part in the research. This provided ‘fresh perception’ of the symptoms from the informants. Men and women were placed in separate focus groups. Patients with psychiatric diagnoses were excluded because they were judged incapable of group participation.

From January to May 2011, five focus-groups (two female and three male) consisting of five to six informants were formed using purposive sampling complemented with theoretical sampling based on emerging concepts, categories and subcategories. We were interested in the typicality of patients in order to compare the findings from a study using typical case sampling with other similar samples. A theoretical sample was based on coding, comparison and writing of memos. We used theoretical sampling to develop the theory with questions raised during the analysis. This also allowed us to propose relationships between categories, fill gaps in the existing data, and bring to light what we did not yet know. Theoretical sampling was carried out by selecting participants and by modifying the questions asked. It helped to clarify uncertainties, test our interpretation, and build the emerging theory [23].

Twenty-three informants (14 men and nine women), aged 27 to 53 years, participated. All informants had lived in the Russian north for several decades and all were people with low income. Eight had previously been in prison (all men), 12 informants suffered from alcohol abuse, and 17 were smokers (three of them women). The educational level was not high: two persons had no school education, 12 had graduated from school (nine years), and nine persons had graduated from college. Only three persons lived with families; all others lived alone. Relatives were referred to as a family in the discussion by those who lived alone. The profiles of the patients were incidental but they matched the criteria of purposive sampling because they represented a typical study population.

Two co-moderators worked as psychologists with the patients before the FGDs in order to create trust. The moderator discussed the process of the FGD with the co-moderators only after the FGDs using a peer debriefing process. The moderator also talked with the informants before the FGDs in order to create trust and to obtain informed consent, thereby resulting in a more trusting environment.

### Procedures

We conducted FGDs using a semi-structured interview guide. This focused on patients’ perceptions, knowledge and health-seeking behaviour related to tuberculosis and its management as well as on obstacles and factors facilitating health care seeking behaviour. The initial interview guide included prompts such as the following:

“Kindly tell me what you know about tuberculosis.”

“Why don’t people go to health services when they have symptoms of tuberculosis?”

“What may stimulate people to go to health care services in case of tuberculosis symptoms?”

“What may be done to motivate people to go much earlier for diagnosis?”

We used purposive sampling to clarify and develop explored concepts and to saturate topics, according to emerging codes and categories [23,24]. Data collection and analysis were done simultaneously. We conducted an initial analysis of each FGD before holding the next one, and if important issues emerged, these issues were explored further in the next FGD [23]. When the next FGD did not bring new information or the results of the next interview could be anticipated, the process of data collection was considered saturated. The writing of memos was done during and after data collection and was important in analysis and sorting. Memos were summaries of major findings and included comments and reflections on particular aspects of our evaluation. Memos also helped to stimulate thought about any additional data.

The FGDs lasted 45–60 minutes and were audiotaped and transcribed verbatim by the principal investigator. We conducted FGDs in the usual environment of the wards of the patients and determined the number and composition of groups according to the ward where they were treated. This was done to follow the guidelines of infection control of the department. Informants, moderator and co-moderators sat in a circle. Patients and researchers used facemasks to prevent transmission of infection. The FGDs were moderated by a principal investigator (a psychiatrist who doesn’t work in the anti-tuberculosis system) and two co-moderators (psychologists who worked in the anti-tuberculosis hospital). The psychologists were a part of the system but the patients were not as acquainted with them as they were with the doctors or nurses. They viewed them as persons who would offer more personal and informal professional interrelationships. The co-moderators took notes and occasionally posed questions but mainly observed the groups. The moderator took notes during and after each FGD, facilitating preliminary analysis.

### Data analysis

**Figure 1:**
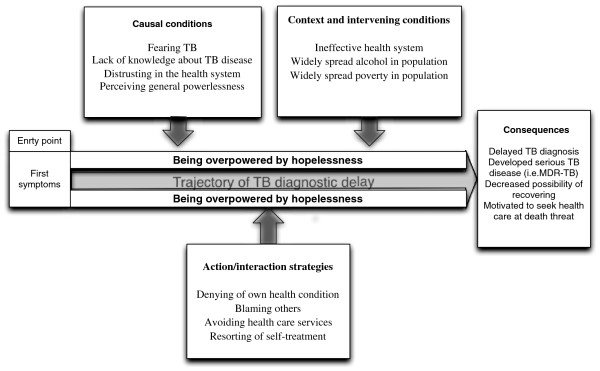
The trajectory of tuberculosis diagnosis delay among patients in Arkhangelsk region.

We carried out data analysis on three levels: open, axial and selective coding [23]. We used open coding, a line-by-line scrutiny of the data, in order to identify the codes expressed by the participants. We labelled related codes and grouped them into categories. Fifteen categories emerged from the data. We identified relationships as illustrated in Figure 1. We conceptualized categories by specifying the relationships between them during the axial coding. We identified a core category, which related to all other categories at the selective coding stage. We used selective and axial coding to knit the concepts of the theory together and generated theory through comparative analysis, which both subsumes and assumes verification, and through accurate descriptions, but only in the frame of generation coding. In using the Paradigm Model, we explored the relationships between concepts [23].

The basic purpose of this model is to enable the researcher to think systematically about data and contextualize the phenomenon by relating categories and concepts in complex ways. The core category that is the central idea, event or happening is defined as the phenomenon. Other categories are then related to this core category according to the schema. This model helps the researcher to integrate all generated categories and concepts based on a systematic approach. Following Strauss and Corbin (1998), we used a coding family that consists of causal conditions, the phenomenon, contextual conditions, intervening conditions, interactional strategies, and consequences [23].

The analyses were carried out by the first author (V.K) in collaboration with the co-authors. Some authors had a previous understanding of the context of tuberculosis care, having been involved in tuberculosis management in the region or/and from previous study of tuberculosis. Being uninvolved in tuberculosis management, V.K was a moderator of the FGDs to explore new ideas and to ensure the discovery of new knowledge. The analysis phase was conducted with members of the research team to compare codes, validate the interpretation of data, and to resolve any discrepancies in the observed findings. Finally, the results were presented to the collaborating hospitals.

We used the principle of theoretical saturation to ensure that all levels of codes were completed and that no new conceptual information was available. We concluded data collection after 5 FGDs.

Ideas different from those of the evolving code list were considered important and given special attention during the analysis. Deviant case analysis was used in considering extreme examples to increase the trustworthiness of the research through showing the context and limits of the emerging theory.

We found two main concerns and core categories in our material and in this article we present the most common one judging by what most participants told us.

### Ethical considerations

The research was approved by the Ethics Committee of the Northern State Medical University (Arkhangelsk). We informed each participant of the purpose of the research and obtained signed consent forms from all participants. Every informant was given the opportunity to refuse to participate in the group discussion at any time without negative consequences. One informant declined to participate in the discussion after being requested to sign the written consent.

Anonymity was maintained by omitting names and personal data. Only the principal investigator had access to names and other confidential information. Participants provided consent that the interviews could be recorded and analysed later.

## Results

The main focus of this study was the manner in which the participants acted and interacted in describing how they handled their situations. The main concern of the patients was that they were overpowered by hopelessness. “Being overpowered by hopelessness” was also identified as the core category. This reflected how they handled their life situations with their symptoms of TB. This core category was interrelated with and influenced by the four causal and three context conditions, which led to the four consequences shown in the model. The second main concern, related only to the “well-being” segment of informants, was having good awareness of the disease. In our research, this group focused on the health system’s delays.

The opinions in the groups were very similar. Some differences between men and women were observed. Men more frequently reported patient delay while women more often reported health systems delay. The model we constructed [23] was named “The trajectory of TB diagnostic delay among patients in Arkhangelsk” (see Figure 1).

### Causal conditions

“Causal conditions” are a set of events that lead to tuberculosis diagnostic delay. Four categories (“fearing TB”, “lack of knowledge about TB disease”, “distrusting the health system” and “perceiving general powerlessness”) were generated (see Figure 1).

Tuberculosis is perceived as a stigmatizing disease. People try to deny the presence of the disease as long as possible but then responsibility to the society and the family is “switched on”. In contrast, stigma plays a minimal role in the family. Some relatives convinced the informants to go to a clinic to seek a diagnosis while some informants went to the clinic because they worried about members of their family. Stigmatization was more visible in the context of work, causing concern about working relationships and future possibility for work.

The following citations illustrate these findings:

It is a shameful disease. It is impossible to tell somebody about it. It is not the same as for cancer, for instance. You can talk about cancer, but not about tuberculosis. The husband of my sister had tuberculosis, we were afraid of associating with him…It is said that tuberculosis is incurable; it may be a hidden disease…It is a terminal illness…(Female FGD)

I had the possibility to get a post…. I could go by bus, but that is not a good thing to do, so I walked there. Before I was diagnosed with tuberculosis, I infected everybody around me in 20 days, it is scary! (Female FGD)

Family life was under pressure because of tuberculosis, but there were two different perceptions. The family supported most of the patients and even pushed them to the health system which was a positive factor for early diagnosis.

Everybody reassures me…Supports me! How could it be otherwise?! (Female FGD)

Very few informants related contradictory experiences.

A woman said that she would not be allowed to stay in her home after she is released from hospital, because she may infect her granddaughter. (Female FGD)

As for relations with friends and other acquaintances, the support depended on the situation and the level of closeness in the relations.

I think people will avoid me! The friends advised me to go to the clinic. They said, you will recover and will be as others. (Female FGD)

Fear of TB interrelated with lack of knowledge about symptoms of tuberculosis. Many informants stated that they were not aware of it as a severe disease when they became ill. Some said that tuberculosis should have ‘special’ symptoms, not those similar to symptoms of a cold.

The following citations illustrate the causal conditions:

It is very hard to discover the person who is the source of infection… You meet at least 100 persons per day… The disease is fatal. It is impossible to discover (the source). (Female FGD)

If I knew that it is tuberculosis I would run to the clinic immediately! (Male FGD)

I did not know that tuberculosis is such: these symptoms were simple; I have had similar ones many times before (Female FGD)

The participants related that people distrust the health system, perceiving it as ineffective because of experiences involving various errors by medical specialists. These errors were a part of the diagnostic process or affected the right diagnosis and treatment. Very often, doctors and medical assistants were described as incompetent. Informants stated that “good doctors” do not go to a village to work and only “alcoholics and incompetent specialists work in the districts”. If a doctor’s assistant was good (in the patients’ opinion), he/she would still be considered as not having enough knowledge because of a lower education than the doctors. This exacerbates the existing lack of facilities in the small health centres.

Several days I was treated for pneumonia, but unsuccessfully. (Male FGD)

They prescribed antibiotics for me…. I took them at home… Then I went to the hospital, they relieved the cough, but not the fever. After half a year I went to the hospital again…. After one month I was discharged…. I have been working and working… I wondered why I had been losing weight? The temperature was 39 degrees. My lymph nodes had become affected… fluorography did not show the disease in my case…. (Male FGD)

The doctor visited me but only prescribed medicines. They did not discover my problem… something wheezed … they gave tablets to me…. I went to the sauna… then I visited the clinic, they said…. Your lungs do not breathe… It was a rib fracture in my case… If you say to the doctors, that it is painful to breathe, they just say: “It is a cold, everything will be OK…” (Male FGD)

General powerlessness refers to the perception that there is no possibility to influence one’s own life or health, which causes a delegation of authority to somebody or something else. Very often, these patients complained about authorities at all levels. This is the position of an observer, not an actor in his/her own life. However, the difference from an observer position is that suffering is “included” as a part of such a life.

Half of the people in the village never make a health check-up. If you catch severe disease, you can do nothing! (Female FGD)

Half of the people in the village are made up of sick persons. Nobody checks themselves. I meet everybody. Some persons have not even enough money for food. (Female FGD)

If people were divided into two groups according to their belief in the possibility of changing their lives, TB patients would belong to the group with lower willpower to change. In this group as well, the willingness might be higher or lower, as some considered that they could change (create good relationships in their family, change workplaces, educate their children, etc.), but others did not. A perception of general powerlessness could also affect the individual’s own health. Some might consider improving their own health, others will not. In terms of health-seeking behaviour, this could range from self-treatment in case of illness to just waiting for relief in an emergency situation.

These attitudes of patients lead to passive behaviour that works against early diagnosis.

### Contextual and intervening conditions

Contextual and intervening conditions create circumstances or problems through which groups of individuals respond by their action/interactions.

Three categories (“ineffective health system”, “widespread alcohol in the population” and widespread poverty in the population”) were identified as the context and intervening conditions influencing the phenomenon (see Figure 1).

The prolongation of the diagnostic period was related to the remoteness of health facilities. Many settlements are situated 50–100 km from any medical specialist in the Arkhangelsk region.

It is 80 km to a doctor…. If somebody cuts off a leg at work, he will surely die…(Male FGD)

These components of ineffectiveness of the health system in real life and in the informants’ perceptions create a complex context for care-seeking behaviour. Moreover, tuberculosis was seen as an obstacle to work and to personal and family relations. All informants described an influence of tuberculosis on important aspects of their life, work, family, and relations with friends. Typically, tuberculosis was an obstacle for work because of the diagnosis or the necessity to follow a very strict regime of taking pills in the anti-tuberculosis dispensary even during the outpatient period of treatment. Consequently, they described the necessity of being absent from work for half a day every day. This period is six months at a minimum, but may last much longer in some cases.

A man went to get a job, but there was a shadow in the lung – he had to go to hospital. Doctors held him for a long time (Male FGD)

I got to this place (anti-tuberculosis hospital). Now I have a note in my medical card. I need to have a check-up twice a year. The employer will say: “You are useless to me!” (Male FGD)

It is quite normal for some people to be stigmatized because of their lifestyle of alcohol abuse and the fact that their social setting might be characterized by alcohol abuse. Alcohol behaviour might be “active” or “passive”: some people live in very closed social settings, but some change company from time to time to have the possibility of drinking together with others. As a result, frequency of drinking changes depending on such conditions, varying from once a month to everyday drinking.

Alcohol abuse is a clear problem for Russian society, and its effect on health-seeking behaviour is very strong. Some persons do not seek care because they are oblivious under the influence of high doses of alcohol. Some of them have no homes, medical insurance, or families, and it is unlikely that such persons have a choice to drink or not.

Drinkers are different. You never know is he is ill or not… Always there is alcohol… All use one glass only…. (Male FGD)

Such alcohol consumption leads to a decreased social status and social motivation of a person and finally to stigmatization.

None of the patients could be described as well off. Moreover, patients have to pay for travel and for hotel accommodations in the central city of the region. The treatment is free-of-charge, but transport fees are high for many citizens, especially for the poor people who suffer from tuberculosis. The time required for the diagnostic process was a delaying factor for many informants because they were not able to stop earning money or maintaining their households. Many of them described how they completed work before going to the health system.

It is a problem with no solution - the level of life is very low. Pensioners and old people are ill they have small pensions. Everything becomes more and more expensive…(Female FGD)

People become weaker when it is impossible to find a piece of bread…(Female FGD)

Our town is small, there is a note in my card, it is impossible to get a job……you don’t take pills because you are at work… You will not get pills. No treatment. What do you do? Work? Maybe… you will get a job, but no treatment or food ration…. I work in a private place. It is necessary to go to the polyclinic to take pills. The workplace is far from the polyclinic. So, I am away from work for half of each day. The employer will say: “Get out!” (Male FGD)

### Action/interaction strategies

Action/interaction strategies are purposeful or deliberate courses of actions, which are taken by individuals or groups in response to events, problems or issues, and which occur under certain conditions. The patients aimed their strategies at preserving their own passive position related to hopelessness. We discovered four categories in the action/interaction strategies component of the Paradigm model (see Figure 1). These categories imply behaviour of persons in order to understand “how” people delay getting a TB diagnosis, e.g., denying their own health condition, blaming others, avoiding health care services, and resorting to self-treatment.

Denying one’s own health condition appeared in the typical phrase: “It won’t affect me!” This led to a lack of motivation to visit a doctor and a low awareness of the danger. Some of the informants had a single experience of TB (a neighbour or a member of the family) or a lot of contacts with TB patients (in a prison or in their neighbourhood). They referred to their present or past experiences of getting infected by TB. This experience did not lead to awareness of danger.

I related with tuberculosis ill people before, but I think never it will touch me! They [ill people] were in another side of life (Female FGD)

I was in prison. There was a lot of tuberculosis; I saw many deaths. (Female FGD)

Blaming others was a typical way for informants to explain the reasons for diagnostic delay. Among the things blamed were the health system or some specialists, situations in their own lives, government, policy, etc. Blaming was used to excuse their own mistakes and passive behaviour.

There is no information about tuberculosis. I found posters in the hospital only. If I had known it before, I would have gone earlier [to doctor], (Male FGD)

The queues to the GP in an outpatient clinic are so long! How can I find time for it!? Moreover, very often they are not clever enough. (Male FGD)

Denying one’s own health condition and blaming others led to avoidance of health care that related to distrust in the health system. Informants were not aware of the severity of the disease, considering it as a ‘simple cold’. Culturally, fever for one to two days is not a reason to seek medical help, but for taking pills only. Cough is ‘normal’ for smokers and it is not unusual to have it for many years. Distrust in the health system led to feeling it was useless going to the clinic.

The fever was just two days and it was not so high. As for cough…I have had a cough for several years. I don’t remember how many exactly. It is usual for smokers, and I have been smoking for 25 years (Male FGD)

What is a medical assistant?! He has no good pills! I called for the emergency services. A young boy appeared. He didn’t even listen to me! I did not go to the doctor…. What for?! You have to pay for everything…. I had a fever of 39.5, but he said: “your thermometer is wrong!” We have two doctors in our village. They drink alcohol all the time! (Male FGD)

Feeling unwell made informants initiate action and people treated themselves, for example, for high fever or cough. The self-treatment period could last until the patient became totally exhausted and ill.

I thought it was a cold, so I took pills… In the spring and fall I always get a cold (Male FGD)

There was a cough … I thought, it’s due to dust… I had a lot of family problems and there was no time to visit the doctor. I have been working for months with such a fever (Male FGD)

Taking pills was a typical behaviour among the informants. They did it based on advice of others or from own experience. Many informants had heat applied in a sauna or using a local heater or mustard plaster.

I went to the sauna to get relief. I heated my chest… I didn’t know that this is dangerous……very fast…I took pills and ran to work…I took pills, and I became better…(Male FGD).

### Consequences

The consequences are outcomes of the action/interaction strategies chosen by the actors. Four categories emerged (including “motivated to seek health care by the threat of death” and “developed serious TB disease (such as MDR-TB)”) related to such consequences as “delayed TB diagnosis” and “decreased possibility of recovering” (see Figure 1).

Some informants checked their health regularly or promptly sought care after the first symptoms. Any deviations from normal feelings were motivators for them. Usually, these persons belonged to the “well-being” segment of informants and had good awareness of the disease and prognosis. In our research, these patients focused on the health system’s delays.

For most informants, only a deadly threat became a real motivator to seek medical help. They could not take care of their home or work due to weakness and their work, lives, and alcohol drinking were interrupted as well. Approximately 30% of those who had a diagnostic delay came to the health system by ambulance (*unpublished data*).

Naturally, the fear of death varied for different people but the principle remained the same. For instance, one informant reported about his experience of being in prison, where he encountered a lot of tuberculosis, but he did not seek help right away when the symptoms appeared. Another person had experienced the death of his brother, but the result was the same.

Some were motivated from inside and were aware of the dangers, others were taken to the doctor by relatives, while still other informants worried about social consequences and had a high level of social responsibility. The levels of motivation varied and could range from low to high.

It is necessary to scare people to understand that this disease is deadly; everybody will run to the clinic! Nobody pays attention before getting sick! I discovered blood in my phlegm…… I remembered movies about the war, just run to the doctor! I was sick but did not allow it to interfere with my life and work. When I could no longer get up from the bed…. (Male FGD)

Moreover, other consequences of the diagnostic delay included the increased risk of developing MDR-TB and the spread of tuberculosis in the society.

Causal, contextual and intervening conditions play their role shortly after the start of symptoms, while consequences are important close to the decision to go to the anti-tuberculosis dispensary (Figure 1).

## Discussion

Strengths of this research included the multi-faceted research team that provided a good level of validity and allowed data triangulation. The FGDs allowed wide scope for understanding the meaning of tuberculosis diagnostic delay because informants facilitated each other during communication [27]. We reached saturation after the fifth FGD, allowing us to create a model to explain the phenomenon of diagnostic delay among patients in the Arkhangelsk region [23]. Limitations of the study included the need to wear facemasks and respirators, which introduced limitations to the face-to-face communication.

The theoretical model of diagnostic delay of tuberculosis diagnosis in Arkhangelsk identified the dominant role of the patients’ “being overpowered by hopelessness”.

This core category of “being overpowered by hopelessness” reflects the passive position of persons in this situation, including the patients’ feelings of inability to change anything in their lives, to get a job, or to receive disability benefits. They blamed the administrative system and the doctors for their problems. “Being overpowered by hopelessness” affected their self-esteem and influenced their entire lives, including family, work and social relations. Therefore, health-seeking behaviour could in this sense be seen as contributing to avoidance of diagnosis and treatment. According to the Illness Behaviour Model [19], the biological, psychological, and sociocultural dimensions interact to explain why and how people respond to somatic changes and seek help. In this model, concepts of the disease-illness distinction, psychological mediation of affect, and sociocultural variables are used [19]. Based on this, we can say that “being overpowered by hopelessness” explains how people see themselves as victims rather than active agents for their own health.

People were aware of the conflict between treatment and work. They described how they were unable to carry out their work or could not get a job. They did not trust doctors partly because doctors were perceived to only help in difficult cases, or because the treatment process was expensive for various reasons. Although the treatment was free, patients were often asked to take tests, for which they had to pay. A similar situation was described in Vietnam, where patients were required to pay for travel, drugs, etc. [34].

We cannot say that “being overpowered by hopelessness” is specific to tuberculosis, but it may be common for specific groups of people in Russia who are at particular risk of tuberculosis. The reasons for it are complex. The most obvious are cultural traditions (such as the Orthodox religion), which promotes determinism with both positive and negative values. The destruction of the religious system in the beginning of the twentieth century led people to a sense of victimization that brought negative images into the life philosophy of ordinary people [35]. This appears in the passive behaviour in many parts of their lives. A faith in a leader is typical for underprivileged people; they are accustomed to shifting the responsibility for their lives to a leader. This shift might be aimed at any kind of leader (political, religious, local, or manager at work), depending on the context. Such people remain passive in everyday behaviour by blaming the leader.

The trajectory model provides new dimensions to several types of tuberculosis diagnostic and therapeutic delay described by other authors, including both patient and health system factors. For example, patient delay, doctor delay, health provider delay, diagnostic delay, and treatment delay [3,36,37]. Socioeconomic development is one of the key features in our study as in many other studies, but these studies do not account for the individual position of patients [38]. The model can encompass all types of diagnostic delay [36].

Contrary to the study of Mfinanga et al., [13] we did not identify women as vulnerable. E. Johansson [36], for example, found that women in Vietnam had a TB diagnostic delay two weeks longer than men and had other treatment because of the patient-doctor encounter. Our results are similar to studies from China that identified risk factors of poverty, low educational level, low awareness and knowledge about tuberculosis, and not having any insurance [10]. Flemming et al. [39] and Shin et al. [40] described alcohol problems as a risk factor for tuberculosis. This is in line with our findings. Similar to Thomas et al. [41], who performed a qualitative study of TB patients with alcohol problems, we found alcohol abuse to be a risk factor for delayed tuberculosis diagnosis. Further, alcohol is often related to a low level of social position and peer pressure plays a huge role in alcohol intake.

Our model can be used as a framework for further FGDs and as an interview guide in further research. It would be interesting to find out from in-depth interviews how the passive position has implications in private life. Such phenomena as patient self-esteem and motivation should be studied as influencing the entire life, including family, work and social relations. It is necessary to understand the ways in which treatment and work are incompatible to develop improved management of tuberculosis. Distrust for doctors needs to be clarified by interviews with doctors.

An important finding of our study is that this model might be suitable in similar cultural contexts and other cultural traditions and could lead to a demand for change.

These findings show that it is important to involve people in early medical examination by explaining that tuberculosis can be cured if they ask for medical care in time. It is possible for a patient to avoid passive behaviour if the patient knows what and how to manage the situation with early symptoms.

## Conclusion

The study allowed us to create a theoretical model of the pathway of delay in tuberculosis diagnosis in Arkhangelsk. The model includes categories of causal conditions, contextual and intervening conditions, action/interaction strategies, and consequences. The core category was “being overpowered by hopelessness” as a life position leading to passive behaviour which was common for our informants. The core category provides an answer to the research question: “Why and how do patients in Arkhangelsk delay tuberculosis diagnosis?” It reflects the views of the participants regarding factors influencing the delay in TB diagnosis.

The model allows the ordering of implications for changes in the social and health system, which should aim at decreasing alcohol consumption and poverty and at improving anti-tuberculosis services by including family in support of patients and by reducing organizational obstacles for coordination of employment with taking drugs in Arkhangelsk. Moreover, it is necessary to inform disadvantaged people about tuberculosis symptoms. These actions can reduce diagnostic delay for tuberculosis patients and improve health-seeking behaviour in the future.

## Competing interests

The authors declare that they have no competing interests.

## Authors’ contributions

All authors contributed to the planning the study, VK and AM collected the data, VK, AG, and EJ analysed the data, and all authors participated in writing the manuscript and approved the final version. DE edited the language in the final step. The authors do not have any conflicts of interest.

## Pre-publication history

The pre-publication history for this paper can be accessed here:

http://www.biomedcentral.com/1471-2458/13/712/prepub
